# Self-Tuning Control of Manipulator Positioning Based on Fuzzy PID and PSO Algorithm

**DOI:** 10.3389/fbioe.2021.817723

**Published:** 2022-02-11

**Authors:** Ying Liu, Du Jiang, Juntong Yun, Ying Sun, Cuiqiao Li, Guozhang Jiang, Jianyi Kong, Bo Tao, Zifan Fang

**Affiliations:** ^1^ Key Laboratory of Metallurgical Equipment and Control Technology of Ministry of Education, Wuhan University of Science and Technology, Wuhan, China; ^2^ Research Center for Biomimetic Robot and Intelligent Measurement and Control, Wuhan University of Science and Technology, Wuhan, China; ^3^ Hubei Key Laboratory of Mechanical Transmission and Manufacturing Engineering, Wuhan University of Science and Technology, Wuhan, China; ^4^ Precision Manufacturing Research Institute, Wuhan University of Science and Technology, Wuhan, China; ^5^ Hubei Key Laboratory of Hydroelectric Machinery Design and Maintenance, Three Gorges University, Yichang, China

**Keywords:** manipulator, PSO algorithm, quantization factor–proportion factor, position control, fuzzy-PID control

## Abstract

With the manipulator performs fixed-point tasks, it becomes adversely affected by external disturbances, parameter variations, and random noise. Therefore, it is essential to improve the robust and accuracy of the controller. In this article, a self-tuning particle swarm optimization (PSO) fuzzy PID positioning controller is designed based on fuzzy PID control. The quantization and scaling factors in the fuzzy PID algorithm are optimized by PSO in order to achieve high robustness and high accuracy of the manipulator. First of all, a mathematical model of the manipulator is developed, and the manipulator positioning controller is designed. A PD control strategy with compensation for gravity is used for the positioning control system. Then, the PID controller parameters dynamically are minute-tuned by the fuzzy controller 1. Through a closed-loop control loop to adjust the magnitude of the quantization factors–proportionality factors online. Correction values are outputted by the modified fuzzy controller 2. A quantization factor–proportion factor online self-tuning strategy is achieved to find the optimal parameters for the controller. Finally, the control performance of the improved controller is verified by the simulation environment. The results show that the transient response speed, tracking accuracy, and follower characteristics of the system are significantly improved.

## Introduction

In order to increase the working capacity of the robot, it must be controlled steadily. Therefore, the positioning control of the manipulator is essential. Positioning control is a point–point control in which a fixed parameter setting is specified. The variables of the joint are able to remain in the desired position, unaffected by torque perturbations and independent of the initial state (Lohic et al., 2018; Tian et al., 2020; Jiang et al., 2021a; Liu et al., 2021a). The manipulator is adjusted by the controller so that its joints reach the desired position specified by the user. The difference between the ideal position of the manipulator and the current given value is used as input to the controller to achieve its positioning control (Cheng et al., 2020; Cheng et al., 2021a; Xiao et al., 2021). Currently, distributed PID control of manipulator joints is widely used in the practical production of industrial robots (Jiang et al., 2019a; Qi et al., 2019; Liu et al., 2021b). Although these feedback controllers are designed on the basis of ignoring inter-joint dynamics coupling, they have proven to be effective in practice (Bai et al., 2021; Chen et al., 2021b). On this basis, domestic and foreign scholars and experts are constantly updating the manipulator positioning control algorithm to continuously improve the manipulator positioning control performance (Zhukov, 2019; Ma et al., 2020; Chen et al., 2021c).

Different environments and different shapes of industrial manipulator structures and drive methods are studied. In response to the current shortcomings of industrial manipulators with large mass, large size, and low-positioning accuracy, a highly integrated control system model with small size, light weight, perfect functionality, and electrical hybrid drive was designed (Liao et al., 2020; Duan et al., 2021). Against the shortcomings of the current manipulator with low-positioning accuracy, an adaptive control system is proposed to improve the positioning accuracy of the manipulator (Sun et al., 2020a; Chen et al., 2021a; Yun et al., 2021). However, research on adaptive control strategies has to progress further yet.

In order to achieve high robustness and high accuracy of the manipulator, a self-tuning PSO-fuzzy PID control method is proposed. Quantization factors–scaling factors in the fuzzy PID algorithm are optimized by PSO. The PID controller parameters dynamically are minute-tuned by the fuzzy controller 1 through a closed-loop control loop to adjust the magnitude of the quantization factors–proportionality factors online. Correction values are outputted by the modified fuzzy controller 2. The transient accuracy, response speed, and robustness are improved. The key contributions in the work are as follows:

1) A self-tuning PSO-fuzzy PID control method is proposed.
2) The controller optimal parameters are found by a quantization factor–scaling factor online self-tuning strategy.
3) The performance of the algorithm is analyzed and verified with experimental.

The remainder part of the article is arranged as follows: in *Related Work Section*, the related work for positioning control strategies is described. In *Establishment of Kinetics Model Section*, a four-degree-of-freedom manipulator is modeled. A self-tuning PSO-fuzzy PID positioning controller was designed and simulated in *Methods Section*. The last section is the *Conclusion*.

## Related Work

Now, more and more intelligent algorithms are used to manipulator control systems (Zhang et al., 2017; Lu, 2018; Jiang et al., 2019b; Huang et al., 2019; Nguyen et al., 2019; Huang et al., 2020; Ozyer, 2020; Sun and Liu, 2020; Cheng et al., 2021; Liu et al., 2021c; Yu et al., 2021). Due to various factor interferences, such as the environment, the robot cannot be positioned accurately, and the positioning error will gradually increase (Sun et al., 2020b; Ozyer, 2020; Liu X. et al., 2021). Against the problem of uncertainty in the motion control of the manipulator, the manipulator is controlled to obtain the desired position by means of a calculated torque method (He et al., 2019; My and Bein, 2020), which improves the systematic robust to a certain extent. RBF neural networks can compensate for external environmental disturbances. A PD + RBF control algorithm is combined, which improves the immunity and robust of the power positioning system (Wen et al., 2016; Huang et al., 2021). An adaptive fuzzy SMC algorithm is proposed to the positioning control problem of articulated robots, and the steady-state convergence is good and has some robustness (Zirkohi and Fateh, 2017). Aiming the interference of internal and external factors on the performance of the robotic arm, a joint trajectory sliding mode robust control algorithm is proposed. It can effectively avoid the system jitter phenomenon. However, there are internal models with external disturbances (Weng et al., 2021; Zhao et al., 2021).

While there are algorithms that can improve on some aspects, there can be limitations. A passive-based control method for single-link flexible robotic arms is proposed. Precise positioning of the linkage end is achieved by a combination of precise joint positioning and linkage damping, but the stability is less than ideal (Jiang et al., 2019c; Jiang et al., 2021b). When the modeling is uncertain and the external disturbance is large and complex, it will lead to the phenomenon of jitter and vibration. By improving the interferer, compensating for external disturbances with feedback, and using neural networks to approximate the errors, the jitter is effectively suppressed, and the response speed and tracking accuracy are improved. However, it is suitable for situations where the system modeling error and external disturbances fluctuate greatly (Feliu et al., 2014; Sun et al., 2021). A motion control algorithm with a non-singular sliding mode saturation function method is proposed by combining a sliding mode variable structure and a BP neural network algorithm (Choubey and Chri, 2021; Yang et al., 2021). It provides accurate and stable control of the motion state of the robotic arm. A sliding mode controller was designed (Li et al., 2019a). Variable gain is incorporated into the controller, thus resulting in a controller with high robust and motion control accuracy. However, it is limited to the joint space.

A complete set of gravity compensation algorithms is proposed. According to the joint moment measurements, the parameters are adjusted in real time to meet the dynamic requirements of each stage of the main dynamic positioning process (Wang, 2020). Combining PD control with preset performance control, a simple PD control structure and a preset performance function based on a logarithmic form error transformation are used to design the robotic arm motion controller. The control algorithm improves the dynamic response performance to a certain extent. To speed up convergence, a PD-type iterative learning control law was devised (Zhao et al., 2018). The gain matrix is modified in real time to shorten the correction interval and overcome the problem of slow convergence of system disturbances, but it is less stable.

In summary, there are numerous ways to improve control strategies at this stage, and positioning control strategies are essential. However, there are still problems with some control strategies that need to be addressed, and further research is needed on universality and robustness. The response time and accuracy of the controller also need to be improved. This article finds the optimal parameters by modifying the fuzzy controller to adjust the size of quantization factor–proportion factor online. In the meantime, the improved solution is simulated and compared with the general solution.

## Establishment of Kinetics Model

### Manipulator Structure

**FIGURE 1:**
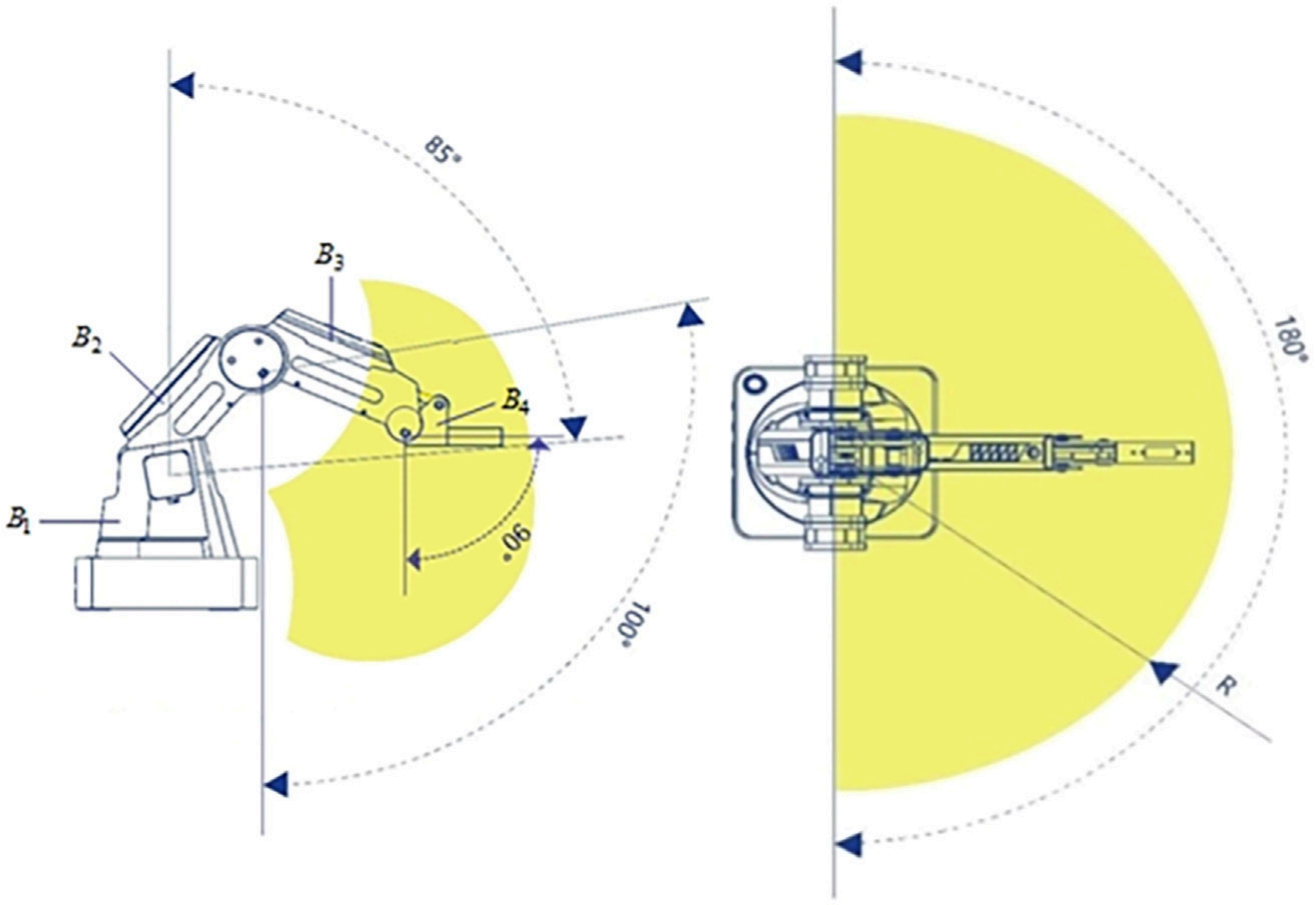
Working space of the four degrees-of-freedom assembly manipulator.

The manipulator working space is illustrated in Figure 1. It has four rotary joints. Combined with the theory, the research will use this manipulator as a carrier to derive a dynamic model.

### Mathematical Model

**FIGURE 2:**
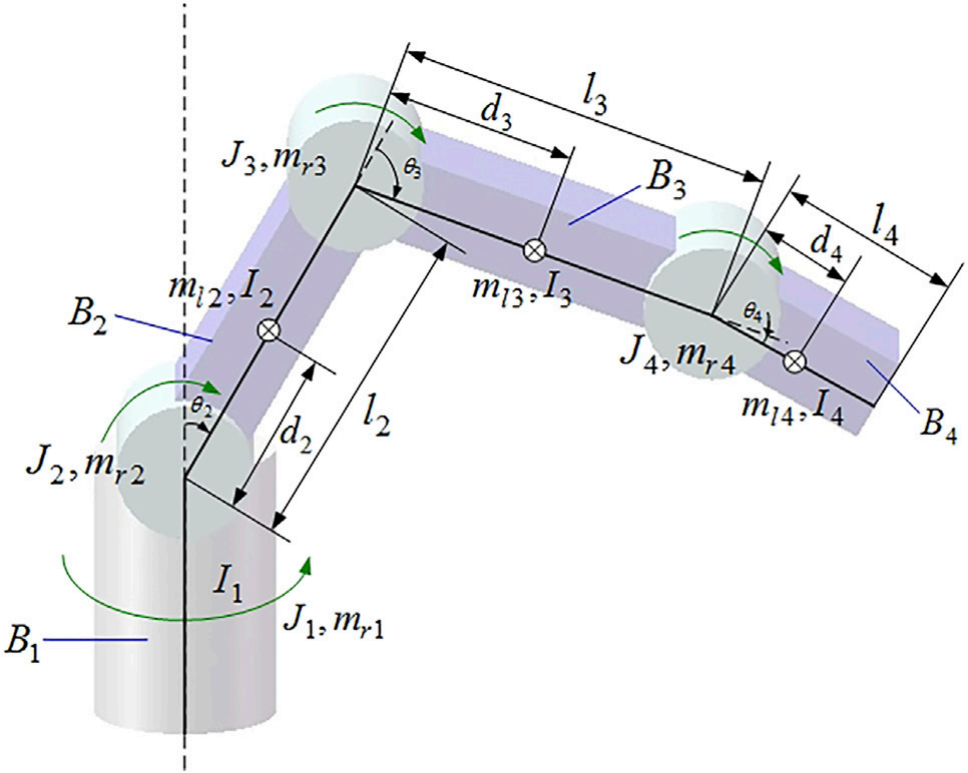
Simplified model of the manipulator.

In order to facilitate the modeling, the dynamic characteristics of the driving circuit, the friction and motion damping between components, and the influence of motor dynamics are ignored (Li et al., 2019b; Ardeshiri et al., 2020; Li et al., 2020; Luo et al., 2020; Garcia et al., 2021). The model simplification of the aforementioned 4-DOF manipulator is shown in Figure 2.

In which, $B_{1}$ is the base, $B_{2}$ is the big arm, $B_{3}$ is the forearm, and $B_{4}$ is the end execution arm. $C_{i}$ is the weight center of the motor rotor, $m_{r,i}$ is its weight, and $J_{i}$ is its moment of inertia. $I_{i}$ is the moment of the connecting rod inertia, $m_{l,i}$ is its weight, and $\theta_{i}$ is its rotation angle. $d_{i}$ is the connecting rod centroid distance and $l_{i}$ is the connecting rod length (Sun et al., 2020c; Yu et al., 2020; Tao et al., 2022). The parameter value set of the manipulator are illustrated in Table 1.

**TABLE 1:** Manipulator parameter values.

Symbol	Meaning	Numerical
[ $m_{l1}$ , $m_{l2}$ , $m_{l3}$ , $m_{l4}$ ]	Link [1, 2, 3, 4] mass ( kg )	[4.30, 7.73, 6.64, 2.01]
[ $I_{1}$ , $I_{2}$ , $I_{3}$ , $I_{4}$ ]	Link [1, 2, 3, 4] rotation inertia ( $kg\cdot m^{2}$ )	[0.045, 0.43, 0.30, 0.009]
[ $m_{r1}$ , $m_{r2}$ , $m_{r3}$ , $m_{r4}$ ]	Motor [1, 2, 3, 4] role mass ( kg )	[0.146, 0.146, 0.042, 0.042]
[ $l_{2}$ , $l_{3}$ , $l_{4}$ ]	The connecting rod is [2, 3, 4] length ( m )	[0.53, 0.39, 0.11]
[ $d_{2}$ , $d_{3}$ , $d_{4}$ ]	Link [2, 3, 4] center of mass distance ( m )	[0.25, 0.18, 0.05]
[ $J_{1}$ , $J_{2}$ , $J_{3}$ , $J_{4}$ ]	Motor [1, 2, 3, 4] role rotation inertia	[ $9.5\times 10^{-6}$ , $9.5\times 10^{-6}$ , $3.0\times 10^{-6}$ , $3.0\times 10^{-6}$ ]
g	Gravity acceleration ( $m/s^{2}$ )	10

The connecting rod movement, part of the kinetic energy affected can be ignored (Liu et al., 2018; Nabavi et al., 2019). Aiming the complexity of the dynamic model, the coupling terms are not considered (Wen et al., 2017; Loucif et al., 2020; Tan et al., 2020), choosing $q={\left[\begin{array}{cccc} q_{1} & q_{2} & q_{3} & q_{4} \end{array}\right]}^{T}={\left[\begin{array}{cccc} \theta_{1} & \theta_{2} & \theta_{3} & \theta_{4} \end{array}\right]}^{T}$ as the generalized coordinate, Eq. 1 is obtained as the simplified dynamic equation:

$$M\left(q\right)\ddot{q}+C\left(q,\dot{q}\right)\dot{q}+G\left(q\right)=U+\mathrm{S,}$$ (1)

where M(q) represents the systematic inertia matrix, $C\left(q,\dot{q}\right)\dot{q}$ represents the systematic centripetal force and Coriolis force vector, G(q) represents the systematic gravity vector, U represents generalized control force vector, and S is the vibration external disturbance force generated. For this model, it has basic properties:

Property 1: It has upper and lower bounds. Scilicet for ∀z vector has Eq. 2:

$$mz^{2}\leq \left\|z^{T}H\left(q\right)z\right\|\leq Mz^{2},$$ (2)

where ‖‖ represents the Euclidean norm of a matrix or vector, 0<m<M .

Property 2: $M\left(q\right)-2C\left(q,\dot{q}\right)\in R^{n\times n}$ , ∀x vector has Eq. 3:

$$x^{T}\left(M\left(q\right)-2C\left(q,\dot{q}\right)\right)x=0.$$ (3)

Property 3: $\exists S_{0}>0$ constant, let the generalized external disturbance force vector satisfy the Eq. 4:

$$\left\|S\right\|\leq S_{0}.$$ (4)

Property 4: $\exists U_{0}>0$ constant, let the generalized control force vector satisfy the Eq. 5:

$$\left\|U\right\|\leq U_{0}.$$ (5)

Property 5: $\exists c_{0}>0$ constant, let the Coriolis force vector and centripetal force satisfy the Eq. 6:

$$\left\|C\overset{\cdot }{q}\right\|\leq c_{o}{\left\|q\right\|}^{2}.$$ (6)

Property 6: The gravitational moment G(q) represents the gravitational potential energy gradient vector $V_{g}$ , scilicet $g\left(q\right)={\left(\partial V_{g}\left(q\right)/\partial q\right)}^{T}$ . $\exists g_{0}>0$ , let the gravity vector satisfy the Eq. 7:

$$\left\|G\right\|\leq g_{0}.$$ (7)

## Methods

### PD Control of Gravity Compensation

The control system instability is easily caused by the integral action in PID. However, as a feedback controller with good closed-loop performance, PD control has extensive applications in single-degree-of-freedom second-order systems (Okamoto and Tsiotras, 2019; Liu et al., 2021d). However, the industrial manipulator with four degrees-of-freedom, the joints and links are not completely independent of each other. There are interactions, so the controllers for each part cannot be designed separately (Sun et al., 2020d; Liu et al., 2021e).

When the controller considers the gravity of the manipulator has the Eq. 8:

$$U_{G+PD}=K_{p}\left(q_{d}-q\right)-K_{D}\dot{q}+G\left(q\right);$$ (8)

$$M\left(q\right)\ddot{q}+C\left(q,\dot{q}\right)\dot{q}+G\left(q\right)=U_{G+PD}.$$ (9)

According to Eq. 8 and Eq. 9, Eq. 10 is the PD control closed-loop equation of gravity compensation:

$$M\left(q\right)\ddot{q}+C\left(q,\dot{q}\right)\dot{q}+K_{D}\dot{q}-K_{p}e_{q}=0,$$ (10)

where $e_{q}=\left(q_{d}-q\right)$ , and the balance point is $y=\left[e_{q}^{T},{\dot{q}}^{T}\right]=0$ .

$$V=\frac{1}{2}{\dot{q}}^{T}H\left(q\right)\dot{q}+\frac{1}{2}e_{q}^{T}K_{D}e_{q}.$$ (11)

From M(q) and the proportional gain matrix $K_{p}$ , the global positive definiteness can be known. Eq. 12 is obtained by taking V :

$$\dot{V}=\frac{1}{2}{\dot{q}}^{T}\dot{H}\left(q\right)\dot{q}+{\ddot{q}}^{T}H\left(q\right)\dot{q}+{\dot{e}}_{q}^{T}K_{D}e_{q}.$$ (12)

For $\forall \dot{q}$ , the derivative of a function that is turned into a semi-negative definite, scilicet:

$$\dot{V}=-{\dot{q}}^{T}K_{D}\dot{q}\leq -\lambda_{\min}\left(K_{D}\right){\left\|\dot{q}\right\|}^{2},$$ (13)

where $\lambda_{\min}\left(K_{D}\right)$ is the smallest eigenvalue of $K_{D}$ .

### Fuzzy PID Controller

The output of the controlled manipulator adjusted by the controller is called the driving torque (Sun et al., 2020a; Liao et al., 2021). Aiming the positioning control, the fuzzy control scheme is designed in this section (Li et al., 2019c). Equation 14 is obtained as the simplified dynamic equation:

$$M\left(q\right)\ddot{q}+C\left(q,\dot{q}\right)\dot{q}+G\left(q\right)=U+\mathrm{S,}$$ (14)

where, $q={\left[\begin{array}{cccc} \theta_{1} & \theta_{2} & \theta_{3} & \theta_{4} \end{array}\right]}^{T}$ is the position vector and $U={\left[\begin{array}{cccc} U_{1} & U_{2} & U_{3} & U_{4} \end{array}\right]}^{T}$ is the output torque vector of the controller.

**FIGURE 3:**
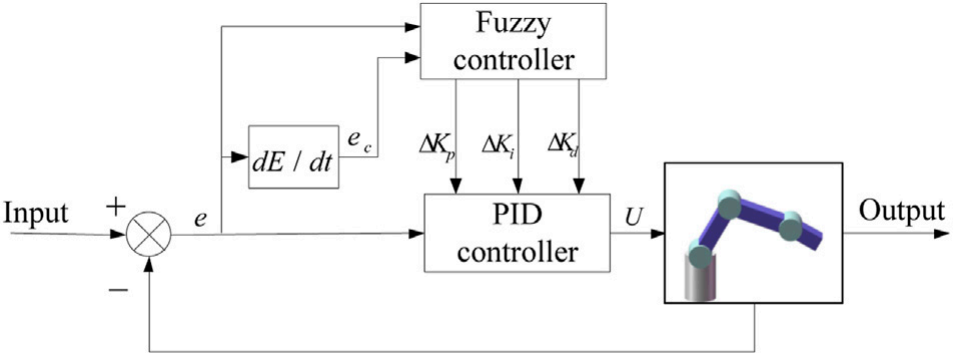
Fuzzy PID control system.

The gravity term of Eq. 14 is moved to the right of the equal sign as part of an uncertain disturbance.

$$U=M\left(q\right)\ddot{q}+C\left(q,\dot{q}\right)\dot{q}-S^{\prime },$$ (15)

where size of $S^{\prime }$ is bounded, $S^{\prime }=S-G\left(q\right)$ , scilicet $\exists {S^{\prime }}_{0}$ is a positive real number so that $\left\|S^{\prime }\right\|\leq S_{0}^{\prime }$ . The hybrid fuzzy PID controller total output is superimposed output of the PD control and the fuzzy controller. Figure 3 is the fuzzy PID control system block diagram of a four-degree-of-freedom manipulator.

According to the dynamic model and characteristics of the manipulator, the structure and parameters of the fuzzy controller are designed. The position deviation and the deviation change rate are the input variables of the fuzzy controller. Expert experience, the fuzzy controller output is obtained after repeated trials. e represents the position error of the manipulator, and $e_{c}$ represents this error rate of change. Their output control volumes are $\Delta K_{p}$ , $\Delta K_{i}$ , and $\Delta K_{d}$ .

e and $e_{c}$ are divided into seven fuzzy partitions. It represents negative big (NB), negative medium (NM), negative small (NS), zero (ZO), positive small (PS), positive middle (PM), and positive big (PB). The conformity degree of elements is quantitatively described by the degree of membership in the domain of discourse M . The fuzzy sets are represented by the membership function. Fuzzy concepts are represented by fuzzy sets. The following definition is proposed:

$$A_{mn}=\left\{x,\mu_{A_{mn}}\left(x\right)|x\in N\right\},\mu_{A_{i}}\left(x\right)\in \left[0,1\right],$$

where $A_{mn}$ is the fuzzy collection of the identification number mn , x is the independent variable of the fuzzy collection, and $\mu_{A_{mn}}\left(x\right)$ is the membership function of the fuzzy partition mn .

**FIGURE 4:**
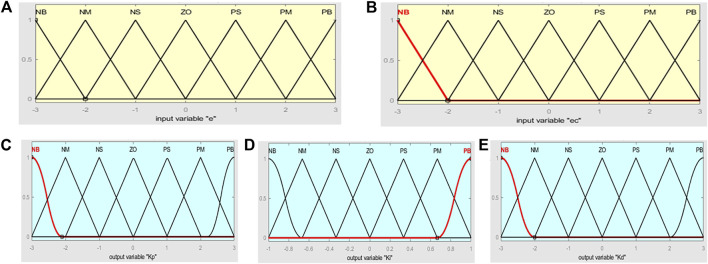
Degree of membership function ( $A\text{:}e,B:e_{c}\text{,}C\text{:}\Delta K_{p},D\text{:}\Delta K_{i},E\text{:}\Delta K_{d}$ ).

Based on the results of multiple simulation, the membership functions of the variables e and $e_{c}$ are adjusted. The triangle membership function is used by them. M represents the domain of discourse. $M_{e}\in \left[-3,3\right]$ , $M_{e_{c}}\in \left[-3,3\right]$ , $M_{\Delta K_{p}}\in \left[-3,3\right]$ , $M_{\Delta K_{i}}\in \left[-1,1\right]$ , and $M_{\Delta K_{d}}\in \left[-3,3\right]$ , and Figure 4 is the degree of membership functions.

**FIGURE 5:**
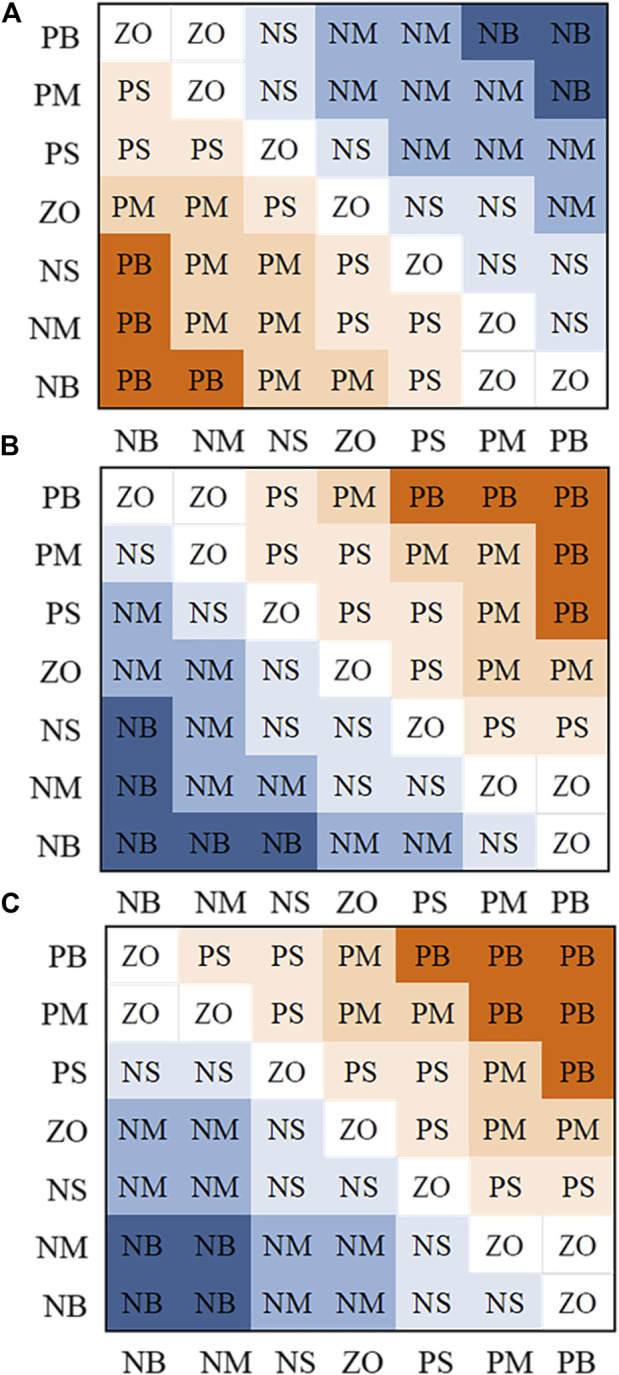
Fuzzy control rule ( $A\text{:}\Delta K_{p},B\text{:}\Delta K_{i},C\text{:}\Delta K_{d}$ ).

According to the positioning control law of the manipulator, 49 fuzzy rules are designed and formed into a fuzzy control rule Figure 5.

**FIGURE 6:**
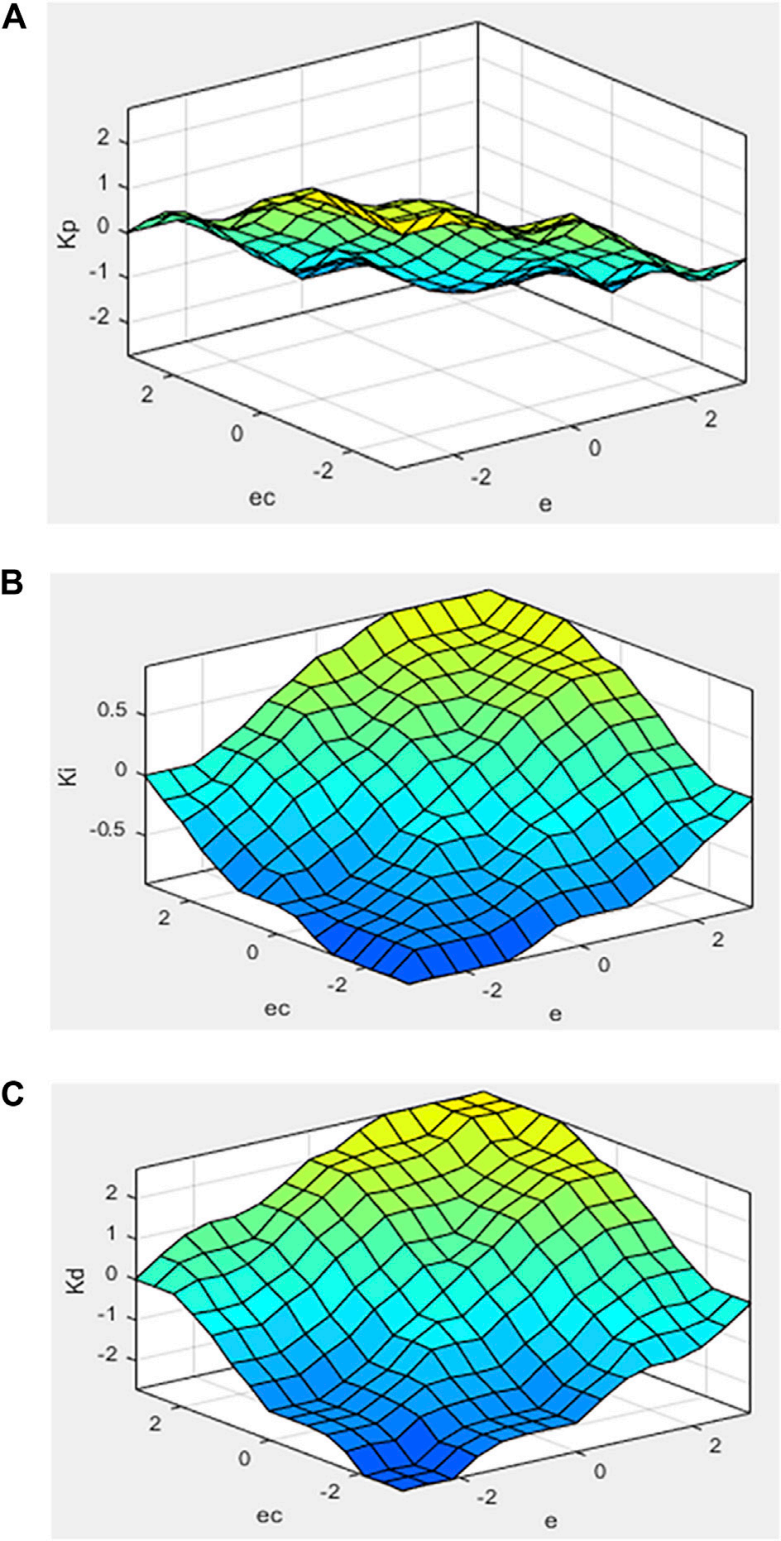
Characteristic face of the fuzzy inference system ( $A\text{:}\Delta K_{p},B\text{:}\Delta K_{i},C\text{:}\Delta K_{d}$ ).

The gravity center method is used for defuzzification, and the Mamdani method is used for fuzzy inference (Fu et al., 2019; Zhou et al., 2019). Figure 6 is the characteristic face of the fuzzy inference system. It can be seen that the output surface obtained is relatively smooth compared to the input.

### Self-Tuning Positioning Controller Design Based on PSO Algorithm and Fuzzy PID

The particle swarm algorithm is a global optimization algorithm inspired by the activity of flocks of birds. Starting from a random solution, iterations are made to find the optimal solution and evaluate quality of the solution by fitness. In the iteration process, the particles update their velocity and position through *Pbest* and *Gbest* to achieve the global optimum.

The update strategy is as follows:

$$V_{id}^{t+1}=W^{t}V_{id}^{t}+c_{1}r_{1}\left(Pbest_{id}^{t}-X_{id}^{t}\right)+c_{2}r_{2}\left(Gbest_{id}^{t}-X_{id}^{t}\right)$$ (16)

$$X_{id}^{t+1}=X_{id}^{t}+V_{id}^{t+1}$$ (17)

$$W=\left\{ \begin{array}{l} W_{\min}-\frac{\left(W_{\max}-W_{\min}\right)\times \left(f-f_{\min}\right)}{f_{avg}-f_{\min}},f\leq f_{avg}\\ W_{\max},f>f_{avg} \end{array}\right.$$ (18)

where, $V_{id}^{t+1}$ and $X_{id}^{t+1}$ are the velocity and position of the particle in the now, respectively. $r_{1},r_{2}\in \left[0,1\right]$ . $c_{1}=c_{1}^{start}+\frac{c_{1}^{end}-c_{1}^{start}}{t_{\max}},c_{2}=c_{2}^{start}+\frac{c_{2}^{end}-c_{2}^{start}}{t_{\max}}$ are the asynchronous learning factor formula. $W_{\max}=0.9,W_{\min}=0.4$ . f and $f_{avg}$ are the adaptation value and the average adaptation value, respectively. The PSO is set to three dimensions.

The process for fuzzy PID based on PSO is as follows:

1) Initialize, run PSO, and retain *Gbest*.
2) The initial number of particles is 100, and the asynchronous learning factor parameters are $c_{1}^{start}=2.5,c_{1}^{end}=0.5,c_{2}^{start}=0.5,c_{2}^{end}=2.5$ . The maximum number of iterations is 100. The setting value of $K_{p}=0.7,K_{i}=0.2,K_{d}=0.9$ .
3) Determining whether the inertia weight is a positive real number.
4) To update the velocity and position of the particle using Eq. 16 and Eq. 17.
5) Evaluate all particles in the population and calculate particle fitness values. The current particle fitness is compared with the historical *Pbest*, and the population all-particle fitness is compared with the historical *Gbest*. In this article, the composite metric ITAE is that the integral of absolute deviation in time, as the fitness; the smaller the value, the better the performance. ITAE can calculate comprehensive assessment of the systematic dynamic and static performance. It is calculated as follows:

$$f_{ITAE}=\int_{0}^{\infty }t\left|e\left(t\right)\right|dt,$$ (19)

where t is time, and e(t) is the control of position error variation.

6) Calculating and updating Gbest.
7) $K_{p},K_{i},K_{d}$ are dynamically adjusted by means of a fuzzy rule.
8) Determine if the termination condition is met. To end when the fitness threshold or maximum number of iterations is obtained, otherwise return to Step 3.

**FIGURE 7:**
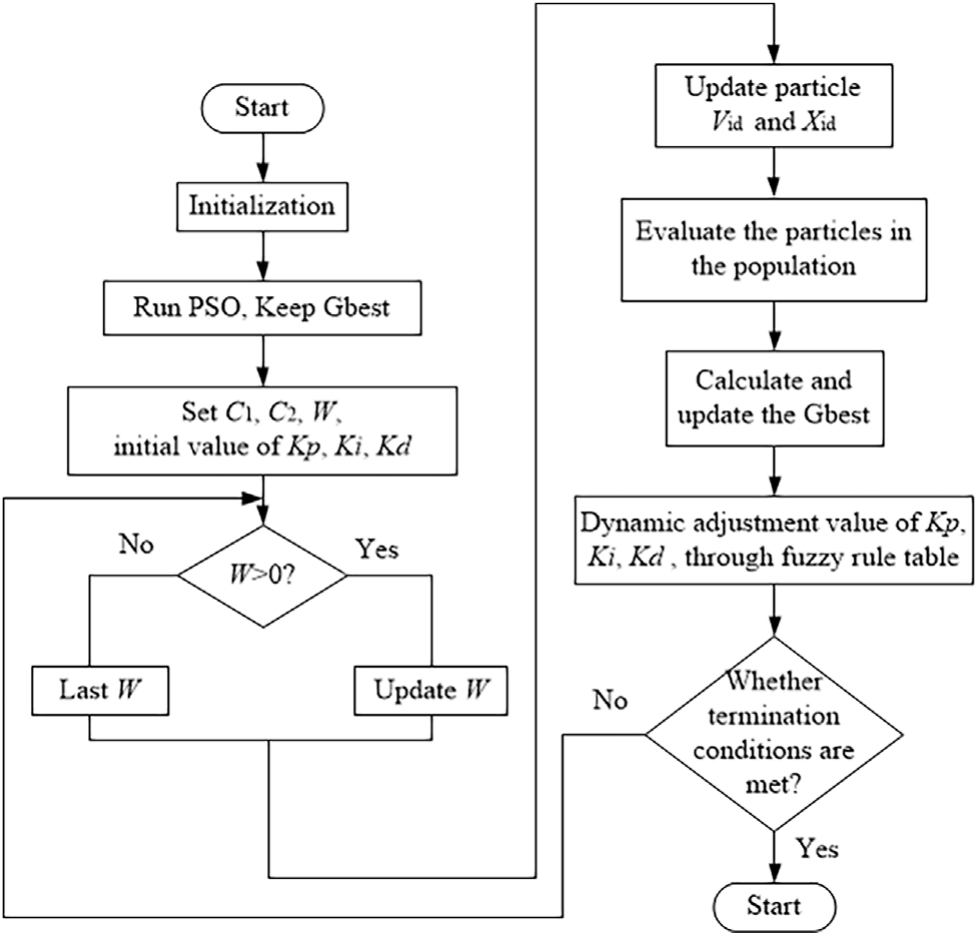
Fuzzy PID algorithm flow based on PSO.

The fuzzy PID algorithm based on PSO is shown in Figure 7.

**FIGURE 8:**
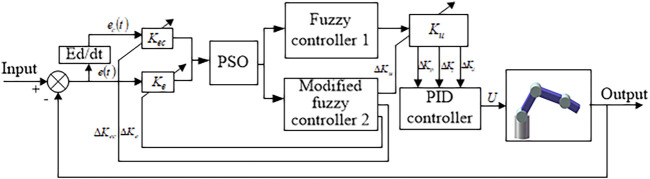
Self-tuning fuzzy PID control system based on PSO.

PID controller deficiencies can be compensated by the fuzzy PID control to a certain extent. However, the quantization factor–proportion factor is usually artificially set. There are some disadvantages, such as difficult to set fuzzy rules, quantization interval, large influence of parameters, and lack of self-adjustment. The article uses a self-tuning PSO-fuzzy PID control method. The quantization factors–proportionality factors in the fuzzy PID algorithm are optimized by PSO. The quantization factor–proportionality factor online self-tuning strategy is adopted as shown in Figure 8.

The quantization–proportion factor controller is constituted of a fuzzy controller 1 and modified fuzzy controller 2. The inputs of the two controllers are the deviation e and the deviation change rate $e_{c}$ . The controller 2 outputs for $K_{e}$ , $K_{ec}$ , and $K_{u}$ are $\Delta K_{e}$ , $\Delta K_{ec}$ , $\Delta K_{u}$ , respectively. According to the controller input changes, the system adjusts the size of the quantization factor–proportion factor online.

Introduce $K_{e}$ , $K_{ec}$ , and $K_{u}$ , and e(t) and $e_{c}\left(t\right)$ are the initial input quantities.

$$e=K_{e}e\left(t\right),e_{c}=K_{ec}e_{c}\left(t\right),u=K_{u}u\left(t\right),$$ (20)

where, $K_{e}$ and $K_{ec}$ become larger, and [−e,e] becomes smaller. e and $e_{c}$ have an enhanced effect on the controller. When $K_{u}$ becomes larger, the basic domain [−u,u] becomes larger, and the role of $K_{u}$ is enhanced (Baigzadehnoe et al., 2017).

The fuzzy rules of $\Delta K_{e}$ and $\Delta K_{ec}$ are determined. Since they are only related to $\Delta K_{e}$ and $\Delta K_{ec}$ , they are divided into {B,M,S,ZO} , which represent {Big, medium, small, zero} . Table 2 is fuzzy rules.

**TABLE 2:** Amendment rules of $\Delta K_{e},\;\Delta K_{ec}$ .

e	NB	NM	NS	ZO	PS	PM	PB
$\Delta K_{e}$	B	M	S	ZO	S	M	B
$\Delta K_{ec}$	B	M	S	ZO	S	M	B

**FIGURE 9:**
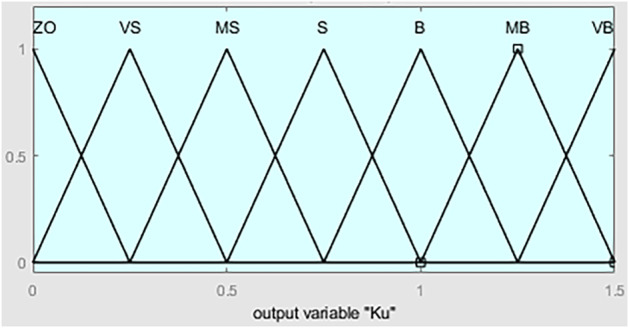
Degree of membership function of $\Delta K_{u}$ .

According to many tests of the conventional fuzzy controller, e and $e_{c}$ are triangular membership function degrees, as illustrated in Figure 9. $M_{e}\in \left[-3,3\right]$ , $M_{e_{c}}\in \left[-3,3\right]$ , and $M_{\Delta K_{u}}\in \left[0,1.5\right]$ .

**FIGURE 10:**
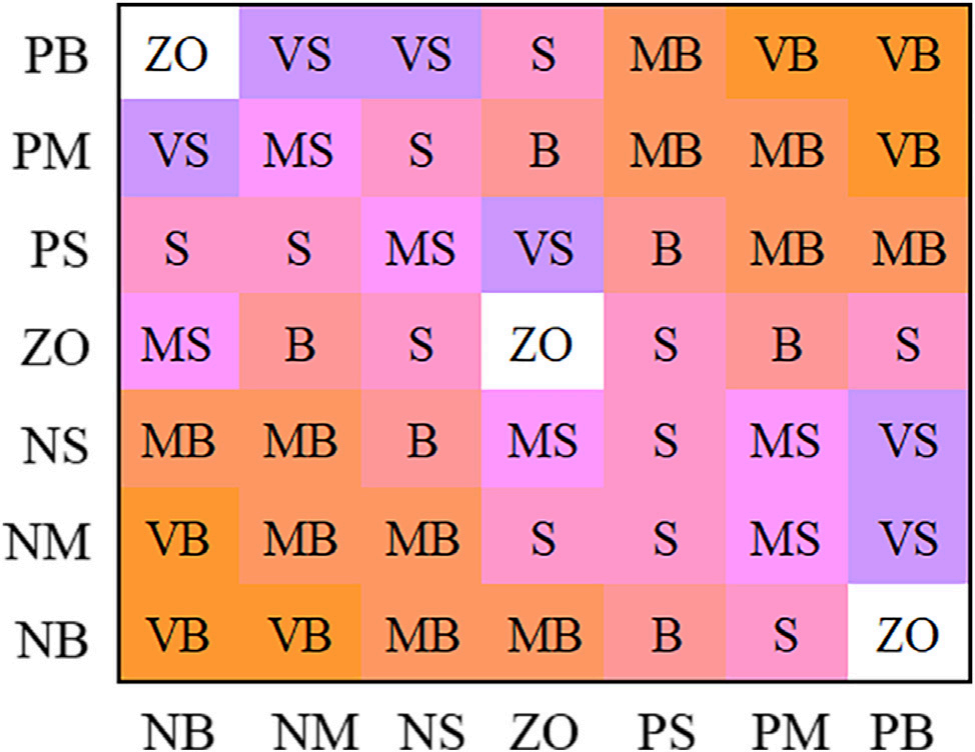
Amendment rules of $\Delta K_{u}$ .

The fuzzy partition of $\Delta K_{u}$ is divided into seven, namely {VB, MB, B, S, MS, VS, ZO}, and they represent {Largest, medium-large, large, small, medium-small, smallest, zero}. Figure 10 is the fuzzy rules.

**FIGURE 11:**
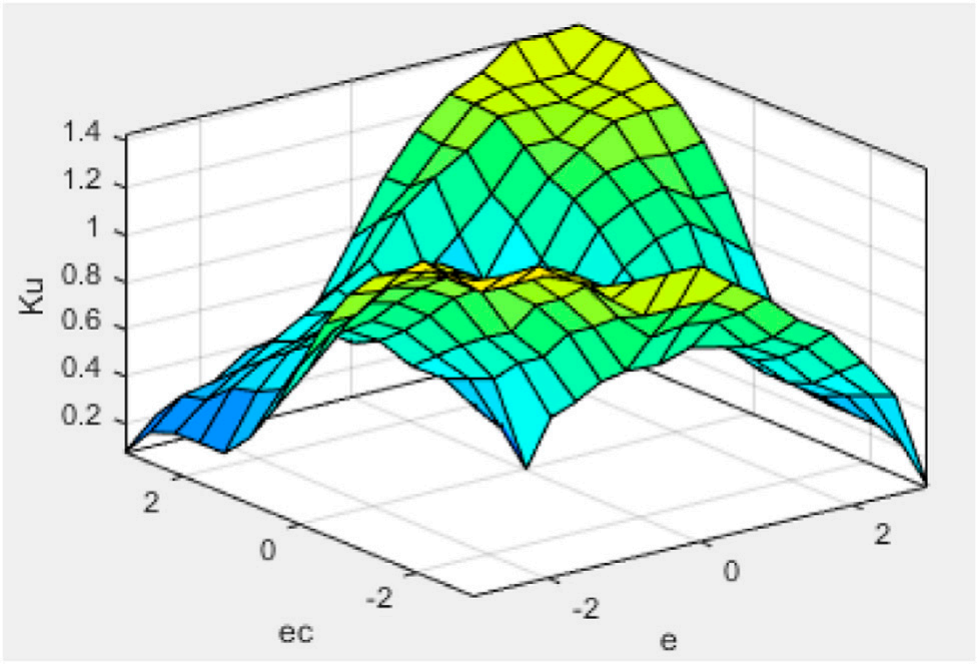
Characteristic face of the $\Delta K_{u}$ fuzzy inference system.

**FIGURE 12:**
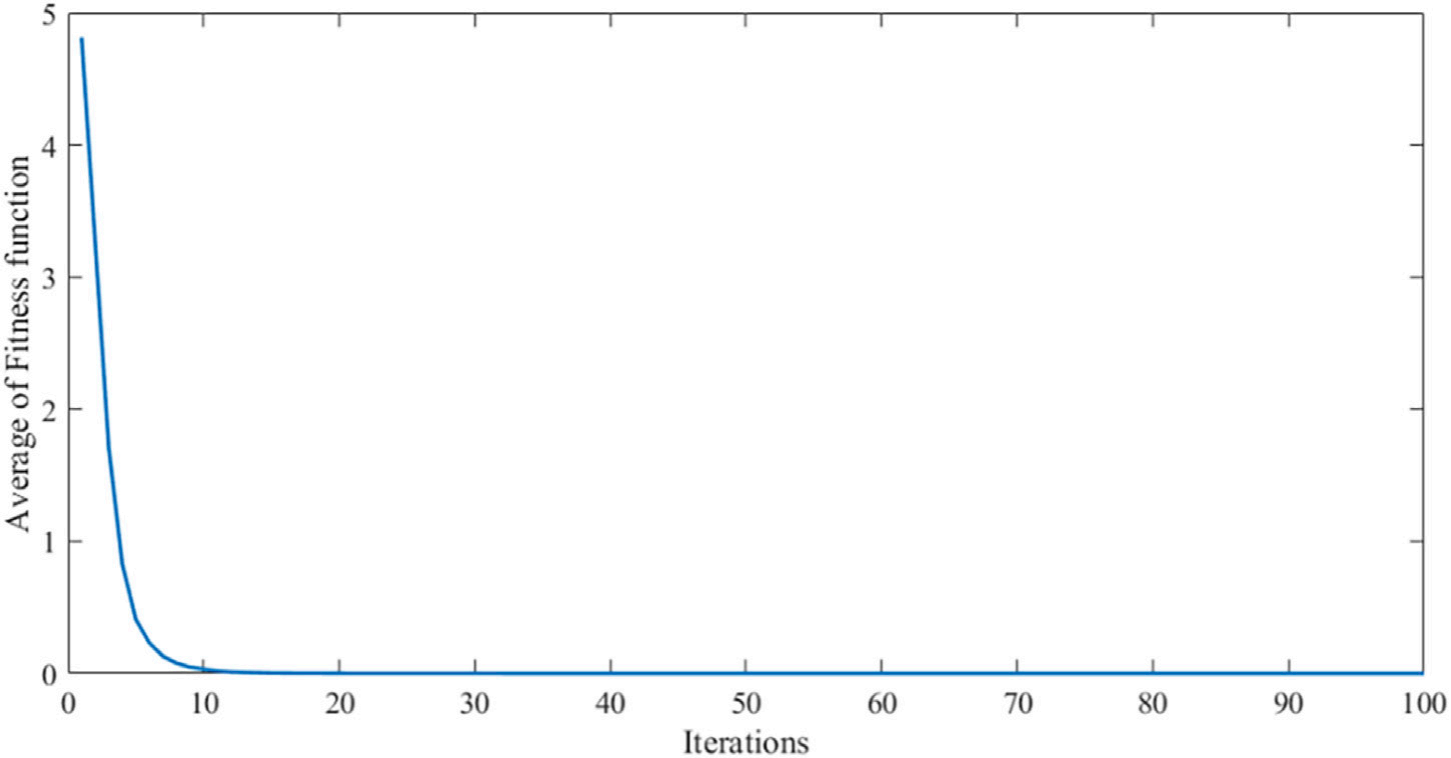
Convergence curve of the algorithm along the function.

All membership function degrees of the modified fuzzy controller 2 are adopted triangular membership function degrees. Defuzzification also uses the center of gravity method. The characteristic face of the fuzzy inference system is illustrated in Figure 11. The convergence curve of the algorithm is shown in Figure 12.

## Experiment

For verifying the effectiveness of the aforementioned controller, a numerical simulation experiment was achieved in MATLAB/Simulink. The real-time state value of the manipulator system is solved by ode4 (Runge–Kutta) (Higueras et al., 2018). Through the feedback of the current state value, the current control torque of the system is obtained. After repeated iterations, the desired state of the manipulator is finally obtained. The solver uses a fixed step size of 0.0001.

The result of the positioning control of the manipulator is verified. The self-adjusting strategy and dual fuzzy controllers are adopted through the PSO-fuzzy PID. The simulation experiment is carried out. It is compared with the conventional fuzzy PID positioning controller.

$q_{1}$ , $q_{2}$ , $q_{3}$ , and $q_{4}$ are the actual angular displacement of joints 1, 2, 3, and 4, respectively. $q_{1d}$ , $q_{2d}$ , $q_{3d}$ , and $q_{4d}$ are the desired angular displacement of joints 1, 2, 3, and 4, respectively. The systematic initial state is set as follows:

$$\left(q_{1},{\dot{q}}_{1},q_{2},{\dot{q}}_{2},q_{3},{\dot{q}}_{3},q_{4},{\dot{q}}_{4}\right)=\left(0.5,0,1,0,0.5,0,1,0\right).$$

**FIGURE 13:**
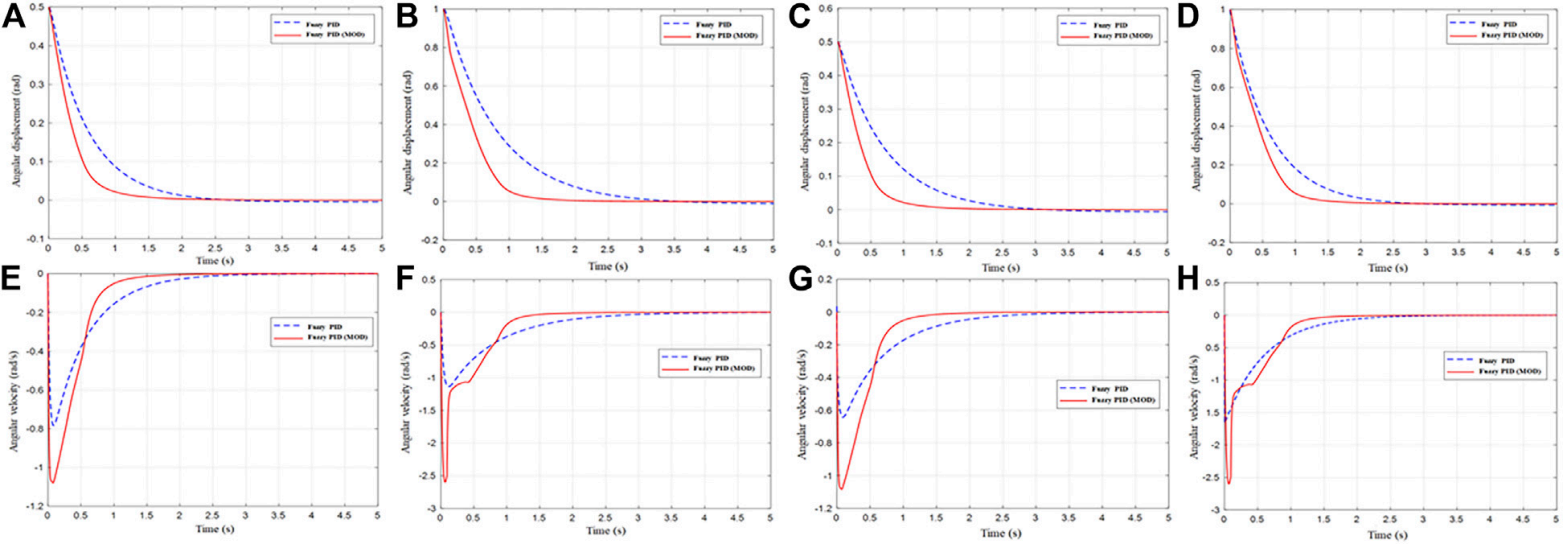
Positioning control response of the angular displacement and angular velocity: **(A)** angular displacement of joints 1, **(B)** angular velocity of joints 1, **(C)** angular displacement of joints 2, **(D)** angular velocity of joints 2, **(E)** angular displacement of joints 3, **(F)** angular velocity of joints 3, **(G)** angular displacement of joints 4, and **(H)** angular velocity of joints 4.

**FIGURE 14:**
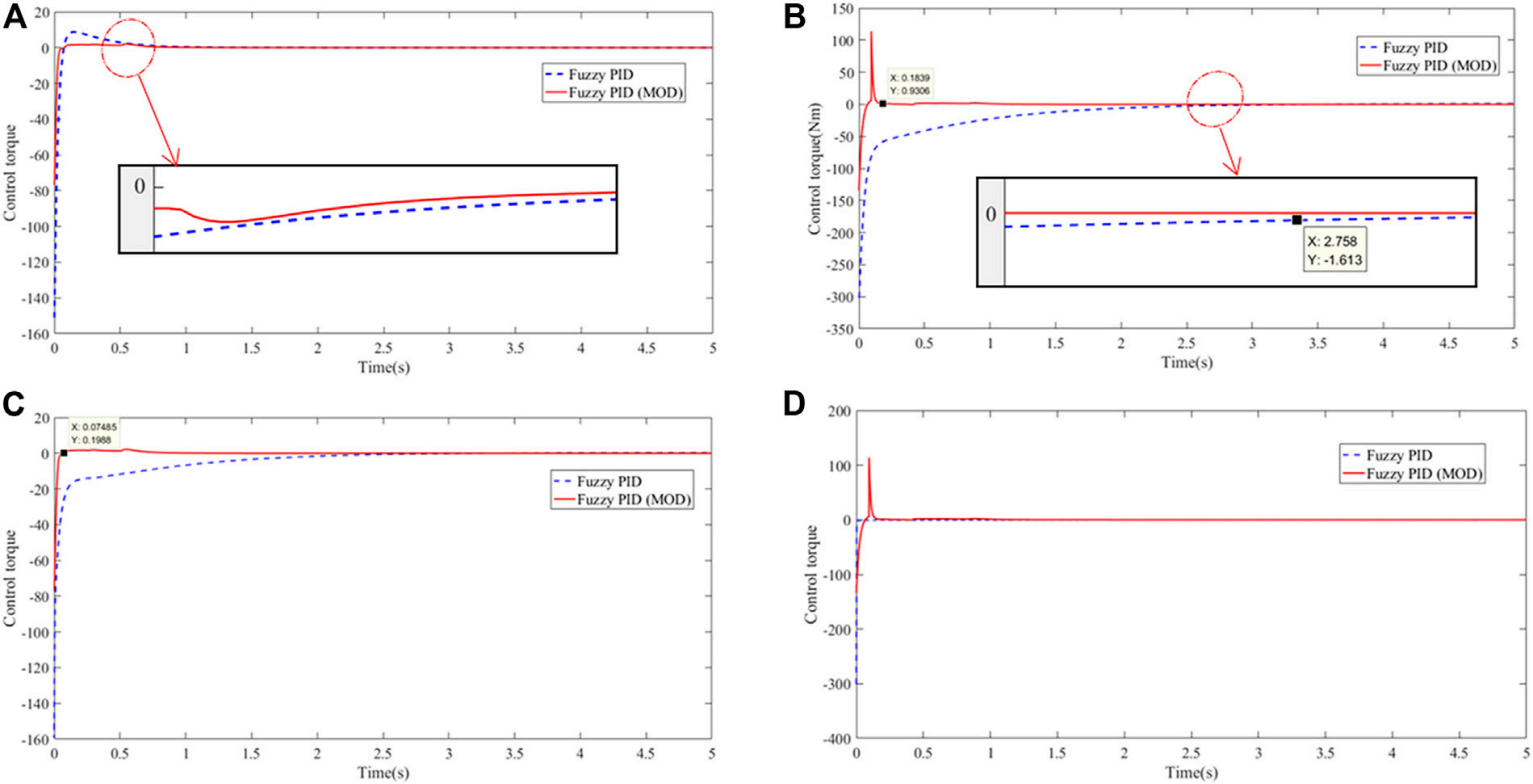
Control torque input curve of the manipulator: **(A)** joint 1, **(B)** joint 2, **(C)** joint 3, and **(D)** joint 4.

The calculation results of the positioning control of joints 1, 2, 3, and 4 are shown in Figures 13, 14:

Under conventional fuzzy controller control, the effective positioning accuracy was set to 0.005. According to the calculation results, the control time of the manipulator was 5s, and the effective positioning time of the joint was 2.8s on an average, with an average error of $7.6\times 10^{-3}rad$ . However, under control of a self-tuning PSO-fuzzy PID controller, the average time for effective positioning of the robot joints was 1.8s, with an average error of $2.8\times 10^{-5}rad$ . The improved self-tuning PSO-fuzzy PID controller has a smoother control input torque than the normal fuzzy PID controller.

## Conclusion

For the positioning control of articulated robots, a modified fuzzy PID controller is designed on the basis of a mathematical model of the manipulator. First of all, the fuzzy control theory is analyzed. Based on empirical data, the type and number of fuzzy subsets of their inputs and outputs and their affiliation functions are designed. Second, the fuzzy set is characterized by an affiliation function curve, and the fuzzy control rules are established. The actual control experience is transferred to the fuzzy controller, and the fuzziness of the output objects is translated into numerical operations. Then, a self-tuning PSO-fuzzy PID positioning controller is designed, and the two positioning controllers are simulated by MATLAB/Simulink. The final simulation results show that the modified fuzzy PID controller has higher control accuracy and smoother control torque than the normal.

The proposed controller can be applied to different industrial manipulators. The future research plan is to apply this controller to the hardware realization of the industrial assembly manipulator.

## Data Availability

The original contributions presented in the study are included in the article/Supplementary material; further inquiries can be directed to the corresponding authors.
